# Adolescents are delayed at inferring complex social intentions in others, but not basic (false) beliefs: An eye-movement investigation

**DOI:** 10.1177/1747021820920213

**Published:** 2020-05-22

**Authors:** Irine Symeonidou, Iroise Dumontheil, Heather J Ferguson, Richard Breheny

**Affiliations:** 1 Department of Linguistics, University College London, London, UK; 2 Centre for Brain and Cognitive Development, Department of Psychological Sciences, Birkbeck, University of London, London, UK; 3 School of Psychology, University of Kent, Kent, UK

**Keywords:** Theory of Mind, adolescence, development, visual-world eye-tracking, executive function

## Abstract

Most developmental research on Theory of Mind (ToM)—our ability to infer the beliefs, intentions, and desires of others—has focused on the preschool years. This is unsurprising as it was previously thought that ToM skills are developed between the ages of 2 and 7 years. Over the last couple of decades however, studies have provided evidence for significant structural and functional changes in the brain areas involved in ToM (the “social brain”) not only during childhood but also during adolescence. Importantly, some of these findings suggest that the use of ToM shows a prolonged development through middle childhood and adolescence. Although evidence from previous studies suggests a protracted development of ToM, the factors that constrain performance during middle childhood and adolescence are only just beginning to be explored. In this article, we report two visual-world eye-tracking studies that focus on the timecourse of predictive inferences. We establish that when the complexity of ToM inferences are at a level which is comparable with standard change-of-location false-belief tasks, then adolescents and adults generate predictions for other agents’ behaviour in the same timecourse. However, when inferences are socially more complex, requiring inferences about higher order mental states, adolescents generate predictive gaze bias at a marked delay relative to adults. Importantly, our results demonstrate that these developmental differences go beyond differences in executive functions (inhibitory control or working memory) and point to distinct expectations between groups and greater uncertainty when predicting actions based on conflicting desires.

## Introduction

Theory of Mind (ToM) describes our ability to infer the beliefs, thoughts, intentions, desires, and feelings of others. It is long known that by 6–7 years old, children’s performance is at ceiling with standardly administered false-belief (FB) tasks (Surian et al., 2007; Wellman et al., 2001; Wimmer & Perner, 1983). Recently, however, paradigms have been developed that involve more sensitive implicit measures, such as reaction times (Apperly et al., 2006; Back & Apperly, 2010; Bradford et al., 2015), brain responses (Ferguson, Cane, et al., 2015; Geangu et al., 2013; Meinhardt et al., 2012), and eye-tracking (Ferguson, Apperly, et al., 2015; Ferguson & Breheny, 2012; Rubio-Fernández & Glucksberg, 2012) where adults’ ToM performance is less-than-ceiling. Despite the creation of more advanced tests of ToM ability, only a few have investigated children beyond the middle childhood years into early adolescence and older adolescents (14 and older).

Looking at studies that focused on younger adolescents, two have used tasks that involved participants’ understanding of belief. The first is a study by Devine and Hughes (2013), which involved a novel ToM reasoning task—the silent film task—and tested children between 8 and 13 years of age. The task is a film-based analogue to the strange stories task (Happé, 1994) that is well suited to exploring the ability to understand others’ beliefs in children beyond the age of 7. Participants were presented with short film clips that depicted scenarios involving deception, FB, and misunderstanding and were tasked with explaining the behaviour of a character. Performance on the task improved with age, independently of individual differences in verbal ability and socioeconomic status, suggesting that ToM abilities do continue to develop through middle childhood.

More recently, Begeer and colleagues (2016) tested typically developing 7- to 13-year-olds as well as children with autism spectrum disorder (ASD) using a modified FB task—the sandbox task—and a visual hindsight bias task. They found that the adolescent control group, as well as the ASD group, showed more bias in a FB condition than in a memory control condition. According to the authors, this suggests that a tendency to prioritise one’s own perspective over the other is more similar among these two groups than previously reported.

Other studies have explored the development of ToM inferences beyond childhood using versions of the director task (Keysar et al., 2000), in which participants are instructed to move objects around a grid by a director who cannot see some objects in the participants’ view (Dumontheil et al., 2010; Epley et al., 2004; Mills et al., 2015; Symeonidou et al., 2016; Zhao et al., 2018). These studies generally converge in showing that the ability to accommodate a speaker’s limited perspective continues to develop between childhood and adolescence to adulthood. Notably, Symeonidou et al. (2016), following Dumontheil et al. (2010), included older adolescents (14–17.9 years) in addition to young adolescents (9–13 years) and adults (19–29 years), which allowed them to track this developmental trajectory more closely. They also included a control condition that required participants to perform the same task in the absence of the director, having been told their instructions would only refer to items on slots without a back panel, controlling for general cognitive demands of the task. As was found in Dumontheil et al. (2010), Symeonidou et al. report that in the director-present condition, but not the control condition, children and adolescents made more errors than adults, suggesting that ToM use continues to improve between adolescence and adulthood. They also found that inhibitory control (IC) accounted for only some of the variance in behavioural results, indicating that it is a factor of developmental change in perspective-taking, but not the only one.

Symeonidou et al. (2016) also collected eye-tracking data during the director task, which allowed them to investigate the online processes that are activated while participants use perspective to identify the mutually available target object. They found that participants who reached for the wrong object adopted a different gaze pattern to participants who reached for the correct object. That is, looking first to the target and then the competitor object *versus* looking first to the competitor and then the target object. Moreover, when considering correct versus incorrect trials separately, the timecourse of bias formation was the same for adults and adolescents. This evidence indicated that participants’ gaze patterns were based on information that is accessed rapidly, while the difference in outcome depended on whether participants adopted the speaker’s perspective.

The version of the director task used in these studies is widely known to be more challenging to adults than other versions (Brown-Schmidt et al., 2008; Hanna et al., 2003; Heller et al., 2008) or than other online FB tasks (e.g., Ferguson, Apperly, et al., 2015; Ferguson & Breheny, 2012; Ferguson et al., 2010). This particular difficulty of the director task has been attributed to the “third” occluded object, which provides the best fit for verbal description from the participant’s own perspective, making it especially difficult to ignore. Indeed, Heller et al. (2016) report that participants show a high degree of “egocentrism” when presented with stimuli from this kind of *triples* director task, but show very un-“egocentric” behaviour when presented with stimuli from *pairs* director task (as in Heller et al., 2008).^1^

Generally, evidence from research that has used various versions of the director task with adults weighs heavily against a model which proposes a strong egocentric-first heuristic (e.g., Back & Apperly, 2010; Keysar et al., 2000, 2003). Heller et al. (2016) propose that multiple representational domains compete in processing communicative stimuli where uncertainty is involved, whereas factors such as goodness of fit of the description in an instruction weight one domain over the others (see also Ferguson & Breheny, 2012). Moving forward, the general question for adult ToM research is what constrains decisions about other agents’ actions. It is widely acknowledged that executive function (EF) abilities are a factor for adults, particularly those for inhibiting salient but irrelevant perspective information (e.g., Bradford et al., 2015; Brown-Schmidt, 2009; Bull et al., 2008; German & Hehman, 2006) or holding information in working memory (WM; e.g., Cane et al., 2017; Carlson et al., 2002; H. L. Davis & Pratt, 1996; Lin et al., 2010; Mills et al., 2015). However, differences in such abilities are not thought to be the only factor, with attention to perspective-relevant situational cues (Brown-Schmidt, 2012; Heyes, 2014; Rubio-Fernandez & Geurts, 2016; Santiesteban et al., 2014), and broader social or cultural factors (Bradford et al., 2018; Ferguson, Cane, et al., 2015; Nielsen et al., 2015; Savitsky et al., 2011; Wu & Keysar, 2007) among the other factors cited as relevant. Results from the adolescent research reviewed above can be seen in this light: Differences between adolescent and adult groups can be attributed to developing EF abilities, and abilities to attend to and process available stimuli that are relevant to mental state inferences. The link between EF abilities and ToM development is in line with research showing that some EF skills, including IC and WM, develop throughout childhood and adolescence, and even into adulthood (e.g., Gathercole et al., 2004; van den Wildenberg & van der Molen, 2004). In this article, we ask whether and how any limitations that are independent of EF abilities might affect adolescents’ abilities to engage in more complex social interactions, requiring richer or higher order mental inferences. In particular, we explored this question by looking at the timecourse of processing more complex situations. As a first step, we revisited the timecourse of processing less complex events.

As far as the application of ToM abilities go, the different versions of the director task are similar to a basic change-of-location FB task. In both cases, the participant has to reason about another agent’s actions given first-order beliefs that differ to their own. To date, ToM in adolescents has been investigated using the *triples* director task. A standard change-of-location FB task does not impose the extra demands of the *triples* director task, due to the role of the better-fit competitor object in the latter (see Note 1). This is borne out by the fact that, in the triples director task, participants of all age groups are liable to make reaching errors, albeit with adolescents and older children making more than adult controls. As mentioned above, Symeonidou et al. (2016) report distinctive gaze patterns for the different behavioural outcomes and that the onset of these patterns occurs at the same time for all groups, very early in the instruction. They conclude that, where adolescents and adults succeed, the timecourse of processing ToM information is comparable. However, this conclusion is based on post hoc assumptions and it also relies on sparse data regarding trials where adults made errors.

One aim of our first experiment is to compare the timecourse of integrating perspective information between adults and adolescents using a task where the outcomes for adults and older children or adolescents are more uniform. For this purpose, we adopted the procedure used in Ferguson, Apperly, et al. (2015). This is a visual-world eye-tracking study in which participants view videos of change-of-location FB or true-belief (TB) scenarios. For adults in this task, gaze bias forms towards the correct location prior to the point of disambiguation and develops in a steady, monotonic way towards that target. Thus, eye-gaze patterns are expected to be more straightforward to interpret than in the triples director task. In sum, we tested whether developmental differences in the timecourse with which adolescents and adults use perspective to predict others’ actions can be detected using a sensitive eye-tracking paradigm that does not require explicit correct/incorrect judgements (cf. Symeonidou et al., 2016). Moreover, the use of a passive “look-and-listen” design means that mental state inferences should be relatively spontaneous, and external demands on executive capacities should be minimal.

## Experiment 1

The task used in the current experiment is adapted from Ferguson, Apperly, et al (2015) and involves videos portraying true- and FB scenarios. Experimental videos presented two characters, Sarah and Jane. At the beginning of the video both Sarah and Jane are standing behind a table which has a target object (e.g., a pen) in the centre and three containers, one on the left, one in the middle, and one on the right. While Jane is watching, Sarah moves the object into one of the three containers. Sarah then moves the object into one of the other containers, either while Jane is still present and watching (TB for Jane) or after she has left (FB for Jane). Participants were then presented with a still image of the final scene of the video while they heard an auditory description such as “Jane will look for the pen in the container on the [left/middle/right].” In a TB condition, the final container would be the object’s true location, and in the FB condition it would be the initial container. During this presentation, participants’ eye-gaze around the visual scene (the three containers) was recorded to examine visual biases to the different containers.

In addition to the main task, we wanted to check whether individual difference measures that have previously been shown to be associated with ToM differed between groups. Here, we tested two measures of EF: IC and verbal WM. Previous studies have found an association between inhibition and the application of ToM inference in children (3- to 4-year-olds in Carlson et al., 2004; 4- to 10-year-olds in Hansen Lagattuta, Sayfan, & Harvey, 2014; 4- to 9-year-olds in Lagattuta, Sayfan, & Blattman, 2010; 2- to 5-year-olds in Nilsen & Graham, 2009; meta-analysis of 3- to 6-year-olds in Devine & Hughes, 2013), in adolescents (Symeonidou et al., 2016; Vetter et al., 2013), and in adults (Bradford et al., 2015; Brown-Schmidt, 2009; Bull et al., 2008; German & Hehman, 2006—though see Humphrey & Dumontheil, 2016). Previous research has also shown a relationship between WM and perspective-taking (Cane et al., 2017; Carlson et al., 2002; H. L. Davis & Pratt, 1996; Lin et al., 2010; McKinnon & Moscovitch, 2007; Mills et al., 2015; Nilsen & Graham, 2009; Schneider, Lam, et al., 2012), with a lower WM capacity or increased WM load leading to poorer performance on ToM tasks. Neuropsychological research has demonstrated that EF has a protracted period of development, which begins in early childhood (~2 years old) and continues into young adulthood, and each subcomponent of EF develops at its own rate (Anderson, 2002; Diamond, 2013; Garon et al., 2008). For example, IC and WM have been shown to develop throughout childhood and into adolescence or early adulthood (e.g., Conklin et al., 2007; Gathercole et al., 2004; Luna et al., 2004; Van den Wildenberg & van der Molen, 2004).

In sum, Experiment 1 aims to (a) replicate the adult findings from Ferguson, Apperly, et al. (2015), (b) examine whether adolescents’ expectations are affected by others’ (false) beliefs as spontaneously and as early as adults, and (c) explore whether individual differences in IC or WM are possible factors in constraining performance during online FB reasoning. In line with previous studies, we also expected to see age-related differences in IC and WM.

### Method

#### Participants

Forty-one participants took part in this study, of which 17 were adults (24–36 years old, *M* = 26.9, *SD* = 2.93) and 24 were adolescents (11–18 years old, *M* = 15.53, *SD* = 2.25). All were native English speakers. The sample size for each group is comparable with the sample size per experiment in Altmann and Kamide (1999), and the total sample matches that used in Ferguson, Apperly, et al.’s (2015) passive group.^2^ Adult participants were recruited from the University College London (UCL) participant pool, whereas adolescents were recruited from London schools. Data from three adolescents were excluded from the analysis due to insufficient eye-tracking data (more than 50% data loss). Thus, the final adolescent group consisted of 21 participants (11–18 years old, *M* = 15.33, *SD* = 2.05). Parents/guardians of all adolescent participants, as well as all adult participants gave informed consent prior to taking part. This study was approved by the UCL Research Ethics Committee.

#### Stimuli and design

##### FB task

The task design and stimuli were based on that used in Ferguson, Apperly, et al. (2015). Thus, the experiment employed a 2 × 2 mixed design, with belief condition (TB vs. FB) as the within-participant factor, and age group (adult vs. adolescent) as the between-participants factor. Stimuli consisted of 16 sets of experimental videos and pictures with their respective auditory description in one of two conditions. The video clips and auditory narratives were taken from Ferguson, Apperly, et al. (2015).

**Figure 1.**
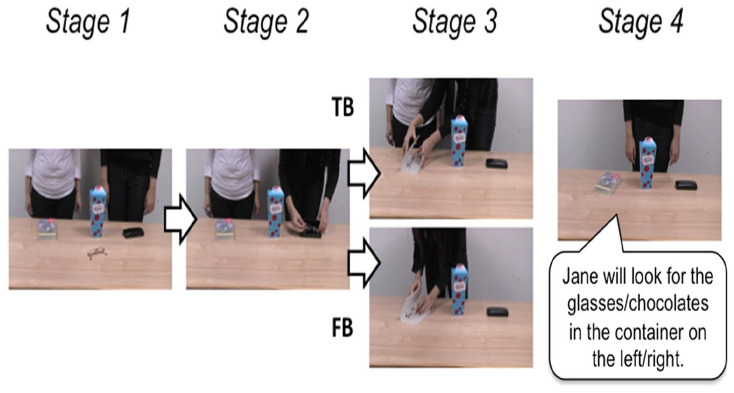
Visual displays depicting key events in the videos during the false-belief task. Stage 1 shows the “start state” where both Jane and Sarah are standing behind the table. Stage 2 was the first part of the video where Sarah moves the target object into one of the three containers. In Stage 3, Sarah moves the object into one of the other containers while Jane was present in true-belief (TB) trials or after Jane had left in false-belief (FB) trials. Finally, in Stage 4 a picture of the “final state” from the video was presented to participants while they listened to the audio sentence.

Two different video sequences set up the two belief conditions (Figure 1). All videos involved a transfer event which began with the two actors (Jane and Sarah) standing behind a table. Videos began with three possible containers on the left, middle, and right side of the table, and the target object in the centre of the table (note that containers and objects changed on each trial). In the first part of the video, Jane and Sarah were both present and Sarah moved the target object into one of the three containers while Jane watched. In the second part, Sarah moved the target object into one of the other containers. Crucially, in one condition, Jane was present for this second transfer event which meant that Jane had a TB about the object’s location. However, in the other condition Jane left the scene after the first transfer events and was absent during the second transfer which meant that Jane has an FB about the object’s location. All videos concluded with Sarah standing alone behind the table and the three closed containers. This final scene of the video was extracted and presented as a picture after the video ended, during which participants heard the following single-pre-recorded sentence: “Jane will look for the [object] in the container on the [left/middle/right].”

One version of each item was assigned to one of two lists and participants were randomly assigned to one of these two lists. Each list contained 16 unique experimental items, eight in each condition (TB and FB). Sixteen filler trials were also included in each list to prevent participants from guessing the aim of the study; videos depicted similar transfer events or the object was replaced into the first container, and auditory descriptions referred to Jane’s, Sarah’s, a stranger’s, or reality perspective (see Ferguson, Apperly, et al. (2015) for full details of fillers). To ensure participants were paying attention, a comprehension question was randomly presented after half of the experimental trials and half of the filler trials (e.g., “The objects are actually in the chocolate box? True < > False”).

##### Individual measures

Participants’ IC and WM were measured. To measure IC, participants completed a simple and a complex Go-NoGo task, with trial type (Go, NoGo) as the within-participant factor. The simple Go-NoGo task was based on the standard Go-NoGo paradigm (Simmonds et al., 2008). A coloured square was presented on the left or right side of the screen in each trial. If the square was green (a Go trial), participants had to indicate which side of the screen it appeared on. If the square was red (a NoGo trial), participants had to inhibit their response. The complex Go-NoGo task used yellow and blue squares and included a one-back WM requirement (see Simmonds et al., 2008). The one-back WM requirement was that participants had to indicate on which side of the screen the square was shown (Go trials) except when a blue square was preceded by a yellow square (NoGo trials). For analysis, we calculated the mean difference in percentage accuracy between NoGo trials and Go trials, and the median correct response time on Go trials, for each participant. Verbal WM was measured using the backward Digit Span subtest of the Wechsler Adult Intelligence Scale–Third edition (WAIS-III; Wechsler, 1997).

#### Procedure

Participants were tested individually and completed the tasks (FB, Go-NoGo, Digit Span) in a single session of approximately 45 min. The FB task was always administered first. Eye movements during the FB task were recorded using a Tobii TX300 eye-tracker at a sampling rate of 300 Hz. Stimuli for the FB task were presented using E-Prime 2 and the Go-NoGo tasks were programmed in Cogent running in MATLAB 7.0 (MathWorks).

For the FB task, participants were given the following instructions as in Ferguson, Apperly, et al. (2015):

In this experiment you will watch short videos, each of which will be followed by a still frame from that video and a spoken description of events. Your task is simply to watch and listen and respond to the comprehension questions when prompted.

After the instructions were given, the experimenter introduced the two characters (Sarah and Jane) by name by showing them a still image of the two characters in the first stage of the video (see Figure 1), with the name of each character written by the character’s side. To ensure that all participants had a clear understanding of which character was Sarah and which character was Jane, they were tested before the start of the experiment by hiding the names from the picture and asking them to point to each character and state their name. Participants were then presented with two practice trials to familiarise them with the task.

A centrally located fixation cross was presented at the beginning of each trial, and the trial was initiated when participants successfully fixated it for at least 1 s without blinking. The video was then presented which showed the transfer events described above. On average, the videos lasted 28 s (ranging from 16 to 53 s). Each video was followed by a 500-ms blank screen before the final state picture from the video was presented. After a 1,000-ms preview of the picture, the relevant audio target sentence was presented. The picture was presented for a total of 7,000 ms. The audio typically ended 1–2 s before the trial ended (see Table 1). After 50% of trials, a comprehension question appeared and participants selected one of two possible answers using the keyboard (all participants achieved at or above 80% accuracy of these questions). Trials were separated by a 500-ms blank screen.

**Table 1.** Average time window durations for each condition (timings in ms).

	TB	FB
“Jane will look for the”	735	740
[object]	868	879
“in the”	210	208
“container”	584	585
“on the”	211	220
[location]	1,002	950

TB: true-belief; FB: false-belief.

#### Eye-tracking data processing and analysis

Participant’s eye movements were tracked while the target image (the “final state”) was on screen and were processed on a trial-by-trial basis relative to the respective image and sound onsets. For each image, three regions of interest (ROIs) were specified around each container location (left, middle, and right). This was done by mapping spatial coordinates of fixations (in pixels) on each ROI, and if a fixation was located within 20 pixels of a container’s perimeter it was coded as a look to that object. Any looks outside these AOIs were coded as background.

The data were then broken down into 20-ms time bins and within each bin samples were binary coded, with “1” belonging to an ROI (Reality, Belief, Distractor, Background) or “0” if there were no looks on the ROIs. These samples were aggregated across participants and items to calculate visual preferences to the *reality location* (the object’s final location) and the *belief location* (the initial location) for seven consecutive time windows of interest. The time windows of interest were (a) the 1,000-ms preview (after image onset but before audio onset), (b) “Jane will look for the,” (c) [object], (d) “in the,” (e) “container,” (f) “on the,” (g) [location]. Table 1 shows the average durations per condition for the word-based time windows.

**Figure 2.**
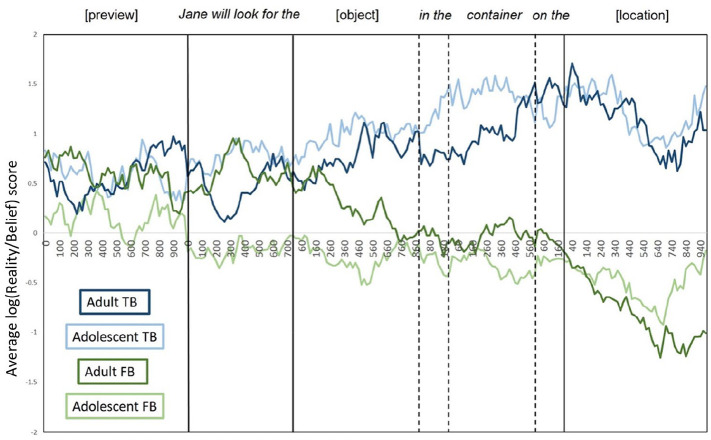
The average log(Reality/Belief) score for each condition and age group. Positive scores show a greater bias to look at the reality location and negative scores show a greater bias to look at the belief location. Note that the dashed and vertical lines indicate absolute onsets and average offsets of individual words in the target sentence.

For each time window, a location-preference score was calculated, as in Ferguson and Breheny (2011, 2012) using: log(Reality/Belief) = *ln, P*_(Reality)_/*P*_(Belief)_. *P*_(Reality)_ refers to the sum of looks to the reality location divided by the total number looks to all ROIs, and *P*_(Belief)_ is the sum of looks to the belief location divided by the total number of looks to all ROIs. The output of this calculation is a single value that measures the bias towards each critical location for each condition within each time window. As this measure is symmetrical around zero, positive scores show a greater bias to look at the reality location and negative scores show a greater bias to look at the belief location. Note that for statistical analysis, eye movements were synchronised to the absolute word onsets and offsets on a trial-by-trial basis. The grand mean for the log-transformed location bias score in each condition and group was plotted to visualise the data (Figure 2), with eye movements resynchronized according to individual word onsets (Altmann & Kamide, 2009; Ferguson, Apperly, et al., 2015).

Statistical analyses were carried out using mixed-effect regression models for each time window separately: (a) [preview], (b) “Jane will look for the,” (c) [object], (d) “in the,” (e) “container,” (f) “on the,” and (g) [location]. The models were fitted using the lmer function in the lme4 package (Bates et al., 2015) using R (R Core Development Team, 2013). The location-bias score, averaged over a given time window, was used as the dependent variable. Each model included belief (FB vs. TB) and age group (adolescent vs. adult) as fixed effects, deviation coded as −0.5 versus 0.5, respectively. Models included the maximal random effects structure suggested by Barr et al. (2013), including random effects for participants and items, and crossed random slopes for belief by group on items and a random slope for belief on participants. Random effects were only removed where they lead to nonconvergence due to overparameterization. Significant effects of context were followed up for each time window using one-sample *t* tests to test whether the fixation bias for each context condition was significantly different from zero (thus indicating *when* participants showed a significant preference to fixate the reality or belief location in each condition). For all tests, a significance level of 5% was used.

### Results

#### Eye-tracking results

Table 2 shows the fixed and random effects for the model adopted in each time window, and Table 3 displays the statistical results from the planned one-sample *t* tests for each condition in each time window where there was a significant effect of condition. Note that as there were no significant interactions between belief and age group, all post hoc *t* tests were collapsed across age groups for each condition.

**Table 2.** Estimates and *t* values for each time window of interest.

	Intercept	Belief	Age group	Belief × Age group
[Preview]				
β Est. (*SE*)	0.72 (0.40)	0.11 (0.22)	−0.36 (0.25)	0.12 (0.44)
*t* value	1.78	0.49	−1.41	0.28
[Pre-object]				
β Est. (*SE*)	0.52 (0.35)	0.52 (0.19)	−0.58 (0.25)	0.71 (0.39)
*t* value	1.47	2.65**	−2.35*	1.84
[Object]				
β Est. (*SE*)	0.65 (0.36)	1.08 (0.21)	0.29 (0.30)	−0.65 (0.41)
*t* value	1.82	5.30***	0.98	−1.59
“in the”				
β Est. (*SE*)	0.41 (0.20)	1.03 (0.18)	0.01 (0.22)	−0.15 (0.35)
*t* value	2.00	5.85***	0.05	−0.43
“container”				
β Est. (*SE*)	0.70 (0.29)	1.72 (0.22)	0.15 (0.31)	−0.51 (0.43)
*t* value	2.93*	7.97***	0.48	−1.19
“on the”				
β Est. (*SE*)	0.55 (0.19)	1.59 (0.30)	0.22 (0.30)	0.38 (0.35)
*t* value	2.84**	9.10***	0.72	1.10
[Location]				
β Est. (*SE*)	0.31 (0.30)	2.79 (0.21)	−0.22 (0.28)	0.39 (0.28)
*t* value	1.03	13.44***	−0.81	0.95

*SE*: standard error.

* *p* < .05; **p < .01; ***p < .001.

**Table 3.** One-sample *t*-test results for each condition and time window where there was a significant effect of condition.

	*df*	*t* value	*p* value
[Pre-object]			
TB	37	3.98	<.001***
FB	37	0.73	.47
[Object]			
TB	37	5.76	<.001***
FB	37	−0.17	.87
“in the”			
TB	37	5.71	<.001***
FB	37	−0.94	.36
“container”			
TB	37	7.47	<.001***
FB	37	−1.00	.32
“on the”			
TB	37	7.59	<.001***
FB	37	−1.30	.20
[Location]			
TB	37	8.76	<.001***
FB	37	−5.01	<.001***

TB: true-belief; FB: false-belief.

*** p < .001.

Analyses showed no significant effects during the 1,000-ms preview period. However, during “Jane will look for the,” a significant effect of belief emerged, reflecting a bias to fixate the reality location when participants shared Jane’s TB about the object’s location but no significant bias to either container when their knowledge of the object’s real location conflicted with Jane’s FB. Interestingly, a significant effect of age group also emerged in this time window, showing that adults experienced a stronger egocentric bias to fixate the reality location across true and FB conditions compared with adolescents.

The significant effect of belief persisted throughout all subsequent time windows. Prior to location disambiguation (i.e., during the [object], “in the,” “container,” and “on the” time windows), this reflected a significant bias to fixate the reality location on TB trials but no significant bias to either container on FB trials. However, once the object’s location was auditorily revealed during the [location] time window, participants showed significant visual biases to the appropriate location (i.e., the reality location when Jane held a TB about the object’s location, and the belief location when Jane held an FB about the object’s location).

To summarise, the eye-movement data suggest that adolescents and adults do not differ in the timecourse of perspective inference and use. Replicating the effects seen in Ferguson, Apperly, et al. (2015), both adults and adolescents showed significantly different visual biases between TB and FB conditions from the pre-object window (“Jane will look for the”), suggesting that participants’ expectations were influenced by contextual information about Jane’s belief as soon as they heard whose perspective to take. However, the significant effect of age in the pre-object time window suggests that adults’ initial processing was more egocentric than adolescents. In addition, both groups showed appropriate biases to the reality location on TB trials in all time windows of interest. However, although participants seem sensitive to Jane’s belief from when they hear “Jane” (i.e., they are not biased to the reality location), they do not seem to use this perspective information until the location time window, where they begin to correctly anticipate reference to the belief location on FB trials.

#### Individual differences results

Results for one-way analyses of variance (ANOVAs) comparing adolescent and adult groups on the individual measures are shown in Table 4. Adults and adolescents did not differ in any of the IC and WM measures (all *p*s > .30).

**Table 4.** Means and standard deviations (*SD*s) of all individual measure scores and their respective one-way ANOVA results.

	Mean (*SD*)		ANOVA	
	Adolescents	Adults	*df*	*F*
Inhibitory control				
Simple Go NoGo (Acc)	−0.08 (0.13)	−0.05 (0.07)	1, 36	0.44
Complex Go NoGo (Acc)	−0.25 (0.14)	−0.23 (0.16)	1, 36	0.19
Simple Go (RT)	385 (46)	382 (23)	1, 36	0.06
Complex Go (RT)	434 (54)	422 (47)	1, 36	0.52
Working memory				
Digit Span	60.91 (2.02)	7.65 (2.34)	1, 36	1.10

ANOVA: analysis of variance; *df*: degree of freedom; Acc: accuracy; RT: response time.

**Figure 3.**
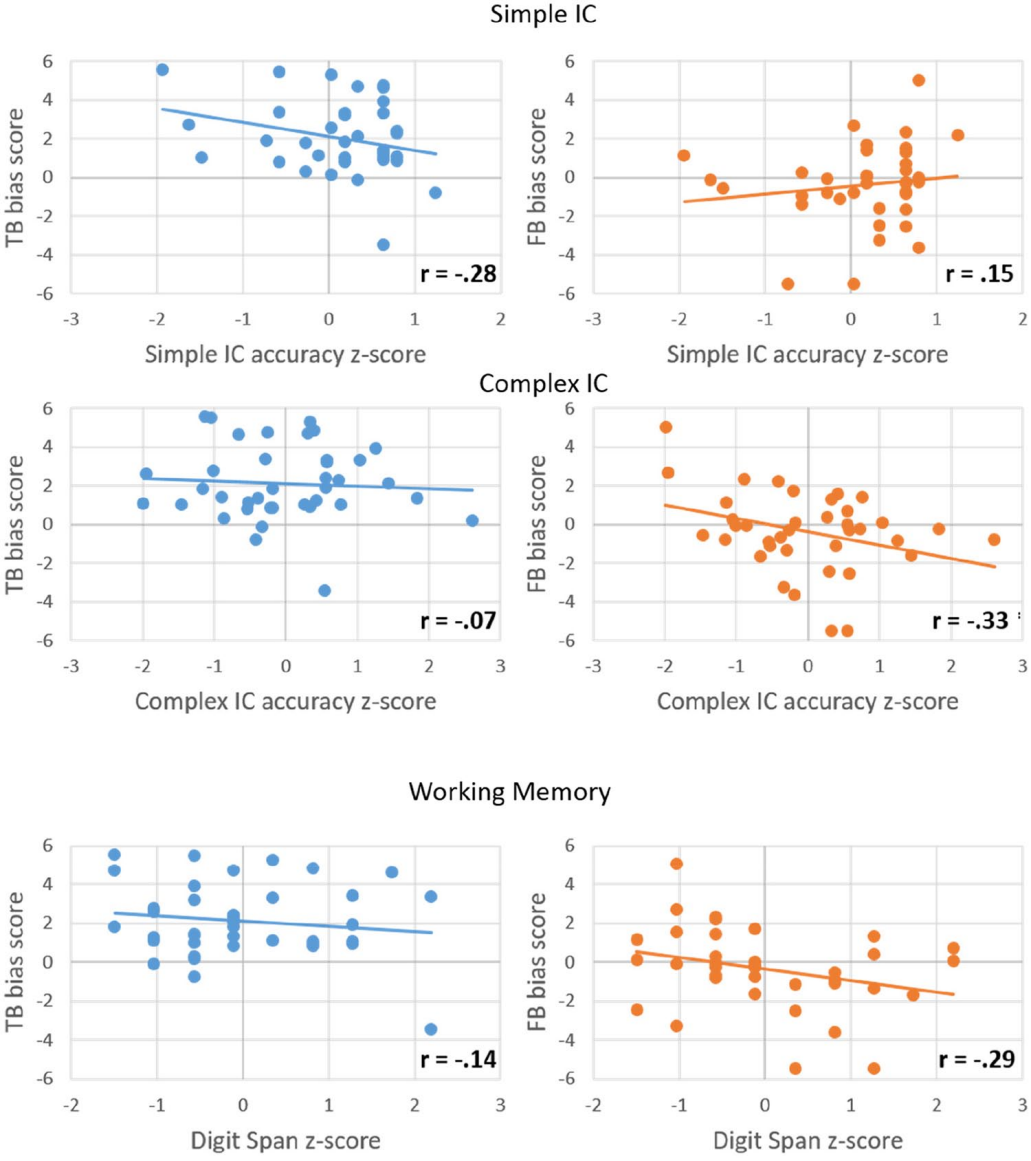
Correlations between participants’ anticipatory bias score (*y*-axis) and each measure of executive function (*x*-axis), separately for the true-belief (TB, blue) and false-belief (FB, orange) conditions. IC: inhibitory control.

To further explore whether individual differences in IC and WM had an effect on looks to the correct target, and whether this differs depending on whether the character’s belief was true or false, Pearson’s correlations were performed separately for each condition between the average bias score during “container on the” and participants’ computed *z*-scores for IC (simple and complex accuracy) and WM. This time window was selected to calculate the averaged bias score as it immediately precedes the disambiguating information, and therefore should show the clearest effects of anticipation. We did not run correlations using the median Go response times since responses on Go trials do not require IC. The significance level for each test was corrected for the six correlations using Bonferroni correction (*p*_crit_ = .0083). The data from these six correlations are presented in Figure 3. Results showed that none of the correlations between anticipatory bias and WM or IC reached significance (all *p*s > .043).

### Discussion

Results from the adult participants in the FB task replicated Ferguson, Apperly, et al. (2015), with participants showing significantly different visual biases between TB and FB conditions from the pre-object window, suggesting an influence of Jane’s perspective as soon as participants heard whose perspective to take. As in Ferguson, Apperly, et al. (2015), adult participants showed appropriate anticipatory looks to the reality location in TB trials. For FB trials, they only made appropriate biases to the initial location when the location was auditorily available. Crucially, adolescents’ data showed the same pattern as adults. Overall, the two age groups’ gaze bias developed in the same way over time, with the adolescents showing a sensitivity to Jane’s belief as early as adults did and appropriately anticipating the reality container in TB trials. Interestingly, a main effect of group during the pre-object window suggested that adults experienced a stronger egocentric bias initially than adolescents. In other words, adolescents suffer less egocentric bias, indicating that not only are adolescents’ performance comparable with adults here, but perhaps may actually be better.

As with the eye-movement data, we did not find any global differences in IC and verbal WM, suggesting that our age groups were well matched on these skills. It is perhaps worth noting that we did not find a significant correlation between complex IC scores and the bias score, even in the FB condition. Such a correlation might be expected given that participants need to inhibit their own knowledge about reality to infer the character’s belief.^3^

In sum, Experiment 1 investigated whether adolescents can reliably infer others’ (false) beliefs as spontaneously and early as adults. This paradigm allowed us to observe the timecourse of predictive gaze in a more straightforward way than in Symeonidou et al. (2016) but confirmed the conclusions of that paper, which is that when perspective information is integrated in predictive inferences, this process occurs in the same timecourse for adolescents and adults.

As discussed above, the director task and the FB task are comparable in terms of the inferential complexity involved; in both cases, an inference has to be made based on a first-order belief of another agent, which differs from the participant’s belief. Therefore, the question remains whether there might a difference between adolescents and adults in a task that involves a greater degree of ToM inferential complexity. A task with greater ToM inferential complexity would require participants to predict or explain another person’s behaviour based on higher order mental states—for example, beliefs about beliefs, desires about what other people believe, and so forth. In Experiment 2, we aim to address this issue by testing adolescents on a task that is arguably less demanding than the FB task in terms of EF demands, but differs in terms of the complexity of the ToM inference. Specifically, Experiment 2 investigates whether adolescents can use knowledge about a character’s higher order preferences about other people’s beliefs about their own preferences (secret desires) to make complex ToM inferences and predict that character’s subsequent behaviour as quickly as adults.

## Experiment 2

In Experiment 2, we adopted the visual-world paradigm from Ferguson and Breheny (2011), where participants were presented with two-sentence stories. The first sentence introduced a property of the character, such as a personal preference, and a context in which either (1a) that character is happy for others to know that property or (1b) the character does not want other people to know about that property. Therefore, in the *open* condition (1a), the basic preference and higher order desires match, whereas in the *secret* condition (1b), the character’s basic preferences and higher order desires are in conflict:

1a Helen does not care who knows that she dislikes vegetables.
1b Helen is very secretive about the fact that she dislikes vegetables.

The second sentence described the character performing an action that is consistent with the context described in the first sentence. For example, in a context such as (1a) Helen would behave in accordance with her personal preferences and appropriately eat something other than vegetables such as meat (2a). In contrast, in a context such as (1b) Helen would adapt her behaviour to fit with her desire to keep personal preferences a secret and would therefore eat vegetables (2b). Participants heard the second sentence while a visual display was presented which included images that were consistent with the character’s two possible choices of action. Although one can predict the appropriate action based on the contextual information given in the first sentence, Ferguson and Breheny (2011) argue that both conditions involve higher order ToM reasoning as the character’s action is not only based on their basic preference but their desires concerning how they want to be viewed by others. Moreover, they observe that the *secret* condition may be more demanding as the character’s basic preference (Helen’s dislike of vegetables) and their high-order intention of keeping this preference a secret are in conflict. This means that since both the basic preference and higher order desires of the character are equally salient, when predicting the character’s action, a participant has to ignore any predictions that are based on the character’s basic preference and they have to make both inferences to make a correct prediction. While in the *open* condition, basic preferences and intentions point to the same target, in the *secret* condition they do not:

2a When Helen goes to dinner parties she makes a show of eating meat.
2b When Helen goes to dinner parties she makes a show of eating vegetables.

The results from adult participants in Ferguson and Breheny (2011) showed that very early on in the linguistic stimulus a difference in visual bias emerged between conditions, despite the fact that the character’s basic preference was the same in both contexts. That is, participants exploited information about both of the character’s higher order desires to predict the target prior to disambiguating information in both conditions. However, the bias in the secret condition was slightly delayed in comparison with the open condition as participants only showed a preference to the target from the post-ambiguous noun region (the offset of “dinner parties” and onset of “makes a show”), suggesting that character’s basic preferences influenced expectations.

The “secrets” paradigm is well suited to address the question raised above because adults’ gaze shows they are successful in rapidly generating predictions using high-order ToM reasoning. In addition, according to Ferguson and Breheny (2011), the task has an advantage over other complex ToM tasks in that it avoids interference from the “curse of knowledge” (Birch & Bloom, 2007). This is a state of affairs in which participants in a task has a more informed perspective than the other person, that is, there is conflicting knowledge from the self-perspective grounded in the actual state of affairs. The secrets paradigm involves stories that require participants to reason about others’ behaviour based on shared knowledge about “reality” and personal preferences, without conflict from the linguistic input or knowledge from the self-perspective. Because the task reduces these demands on IC and other executive mechanisms while involving more complex, higher order ToM inferences, we can explore the development of ToM abilities from a different perspective to the director task and the FB task. As in Experiment 1, participants’ IC and verbal WM abilities were measured. These measures will not only control for individual differences in EF abilities, but they will be informative about our assumptions concerning the kind of demands the secrets task imposes.

### Method

#### Participants

Fifty-two participants took part in this study, of which 17 were adults (24–36 years old, *M* = 27.32, *SD* = 3.57), 18 were older adolescents (14–18 years old, *M* = 16.70, *SD* = 1.39), and 17 were younger adolescents (9–13.9 years old, *M* = 11.81, *SD* = 1.43). Of these, 29 participants also took part in Experiment 1 (11 adults, 12 older adolescents, and 6 younger adolescents), in a separate testing session. Younger participants were included in this experiment than in Experiment 1 to allow us to investigate whether developmental differences may be limited to late childhood/early adolescence. The sample size for each group is comparable with the sample size per experiment in Altmann and Kamide (1999), and the total sample size exceeds that used in Ferguson and Breheny (2011).^4^ All were native English speakers and were recruited and gave informed consent in the same manner as Experiment 1.

#### Stimuli and design

##### Secrets task

The task design and stimuli were based on that used in Ferguson and Breheny (2011). The experiment employed a 2 × 3 mixed design, with context (open vs. secret) as the within-participant factor, and age group (adult vs. older adolescent vs. younger adolescent) as the between-participants factor. Stimuli consisted of 16 sets of experimental pictures with their respective auditory description in one of two conditions and were adapted from those used in Ferguson and Breheny (2011).

**Figure 4.**
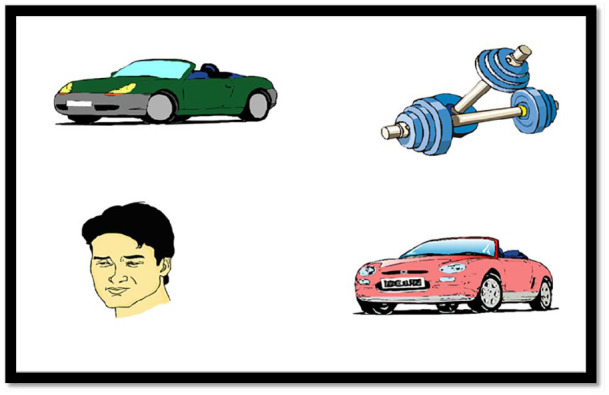
Example visual stimulus used in Experiment 2.

Table 5 shows an example of the experimental auditory stimuli and Figure 4 shows the corresponding visual display. Each visual stimulus contained four images: Character, Open referent (pink car), Secret referent (green car), and an irrelevant distractor object. The position of the four different types of pictures differed across items to prevent participants from creating viewing strategies. The auditory stimulus for each item consisted of two sentences. In the first sentence, a fact about the story character (e.g., *Tom’s favourite colour is pink*) was introduced within an open or a secret context. Second sentence described an event (e.g., *buying a new car*) and referred to an object that was consistent with the character’s open or secret intentions. All character’s actions described in the second sentence were consistent with their higher order intentions (i.e., to be open/secretive about their personal preferences).

**Table 5.** Examples of experimental sentences used in Experiment 2.

Open
Tom is always telling people that his favourite colour is pink
Last week Tom bought a new car and he deliberately chose a pink car.
Secret
Tom does not want anyone to know that his favourite colour is pink.
Last week Tom bought a new car and he deliberately chose a green car.

One version of each item was assigned to one of two lists and participants were randomly assigned to one of these lists. Each list contained 16 unique experimental items, eight in each condition (open and secret context). Sixteen filler trials, using similar visual stimuli but no mental state inference, were randomly distributed in each list. Half the trials were followed by a binary comprehension question to ensure participants were paying attention (e.g., “What type of vehicles were pictured? Convertibles < > Trucks”).

##### Individual measures

As in Experiment 1, IC was measured through a simple and complex Go-NoGo task (accuracy and median correct response time), and verbal WM was measured using the backward Digit Span subtest WAIS-III (Wechsler, 1997).

#### Procedure

Participants were tested individually and completed the tasks (secrets, Go-NoGo, Digit Span) in a single session of approximately 45 min. The secrets task was always administered first, and procedures for presenting stimuli and recording eye movements were the same as in Experiment 1.

For the secrets task, participants were given the following instructions as in Ferguson and Breheny (2011): “In this experiment you will hear short spoken passages and during the second sentence a picture will also be displayed. We are interested in how the pictures help you understand the spoken language.” A centrally located fixation cross was presented at the beginning of each trial, and the trial was initiated when participants successfully fixated it for at least 1 s without blinking. The fixation cross remained on the screen while participants heard the first sentence. The target picture was then presented, and after a 1,000-ms preview the relevant audio for Sentence 2 was initiated. The picture remained on screen for 9 s and Sentence 2 ended approximately 1–2 s before the end of the trial.

#### Eye-tracking data processing and analysis

Participant’s eye movements were tracked while the target picture was on screen and were processed on a trial-by-trial basis relative to the respective image and sound onsets. For each image, four ROIs were specified around each object (Character, Open referent, Secret referent, and Distractor). Any looks outside these areas were coded as background.

The data were then broken down into 20-ms time bins and within each bin samples were binary coded as “1” if they belonged to an ROI and “0” if there were no looks on the ROIs. These samples were aggregated across participants and items to calculate visual preferences to the *open referent* and the *secret referent* for five consecutive time windows: (a) [ambiguous noun] (e.g., “car”), (b) [post-ambiguous noun], (c) [adverb] (e.g., “deliberately”), (d) [transitive verb] (e.g., “choose”), and (e) [disambiguating noun] (e.g., “pink”). Table 6 shows the average durations per condition for the word-based time windows.

**Table 6.** Average time window durations for each condition (timings in ms).

	Open	Secret
[ambiguous noun]	534	541
[post-ambiguous noun]	480	502
[adverb]	825	836
[transitive verb]	597	612
[disambiguating noun]	1,109	1,081

**Figure 5.**
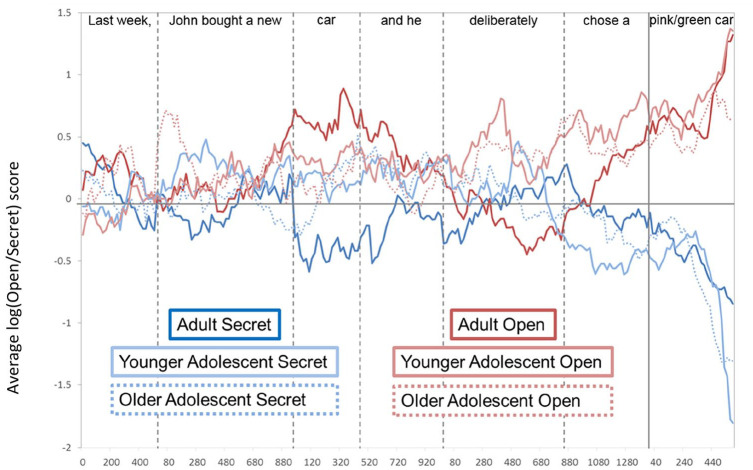
The average log(open/secret) score for each condition and age group. Positive scores show a greater bias to look at the open referent and negative scores show a greater bias to look at the secret referent. Note that the dashed and vertical lines indicate absolute onsets and average offsets of words in the target sentence.

For each time window, a referent-preference score was calculated, as in Experiment 1 using log(Open/Secret) = *ln, P*_(Open)_/*P*_(Secret)_. *P*_(Open)_ refers to the sum of looks to the open referent divided by the total number looks to all ROIs, and *P*_(Secret)_ is the sum of looks to the secret referent divided by the total number of looks to all ROIs. The output is symmetrical around zero, with positive scores suggesting a higher proportion of looks to the open referent, and negative scores indicating a higher proportion of looks to the secret referent. Note that for statistical analysis eye movements were synchronised to the absolute word onsets and offsets on a trial-by-trial basis. The grand mean for the log-transformed referent bias score was plotted to visualise the data (Figure 5), with eye movements resynchronized according to individual word onsets (Altmann & Kamide, 2009; Ferguson, Apperly, et al., 2015).

Statistical analyses were carried out with mixed-effect regression models for each time window separately: (a) [ambiguous noun], (b) [post-ambiguous noun], (c) [adverb], (d) [transitive verb], and (e) [disambiguating noun]. The models were fitted using the “lmer” function in R, and the referent-bias score was the dependent variable. Each model included context (Open vs. Secret) as a fixed effect, deviation coded as 0.5 versus −0.5, respectively. Age group was also a fixed effect and was entered using two deviation coded contrast schemes: Contrast 1 = Adult (1), Older Adolescent (−0.5), Younger Adolescent (−0.5); Contrast 2 = Adult (0), Older Adolescent (0.5), Younger Adolescent (−0.5). This contrast coding allowed us to compare the adult group with both adolescent groups together (Contrast 1) and compare the older adolescent group with the younger adolescent group without the adult group (Contrast 2). Where post hoc analyses were required to follow up on significant interactions, models were re-levelled to include the condition of interest as the reference level. Models used the maximal random effects structure, including random effects for participants and items, and crossed random slopes for context by group on items and a random slope for context on participants. Random effects were only removed where they lead to nonconvergence due to overparameterisation. As in Experiment 1, significant effects of context were followed up for each time window using one-sample *t* tests to test whether the fixation bias for each context condition was significantly different from zero, which would indicate *when* participants showed a significant preference to fixate the open or secret referent. All tests used a significance level of 5%.

### Results

#### Eye-tracking results

Table 7 shows the fixed and random effects for the model adopted in each time window. Table 8 shows the statistical results from the planned one-sample *t* tests for each condition in each time window where there was a significant effect of condition.

**Table 7.** Estimates and *t* values for each time window of interest.

	Intercept	Context	Age group 1: Adults vs. Adolescents	Age Group 2: Young vs. Older adolescents	Context × Age Group 1	Context × Age Group 2
Ambiguous noun						
β Est. (*SE*)	0.17 (0.14)	0.35 (0.18)	−0.16 (0.14)	0.10 (0.24)	0.57 (0.26)	−0.14 (0.46)
*t* value	1.27	1.95	−1.13	0.40	2.23*	−0.32
Post-ambiguous noun						
β Est. (*SE*)	0.09 (0.12)	0.13 (0.17)	−0.06 (0.13)	−0.08 (0.22)	0.38 (0.25)	0.40 (0.43)
*t* value	0.80	0.73	−0.47	−0.36	1.55	0.92
Adverb						
β Est. (*SE*)	0.13 (0.17)	0.34 (0.20)	−0.30 (0.16)	0.01 (0.27)	−0.21 (0.28)	0.18 (0.50)
*t* value	0.81	1.70	−1.91	0.01	−0.74	0.36
Transitive verb						
β Est. (*SE*)	0.13 (0.18)	0.64 (0.19)	0.01 (0.18)	−0.18 (0.30)	−0.51 (0.27)	0.63 (0.50)
*t* value	0.74	3.33***	0.02	0.58	−1.87	1.29
Disambiguating noun						
β Est. (*SE*)	0.04 (0.15)	1.93 (0.19)	0.08 (0.13)	0.13 (0.23)	−0.23 (0.26)	0.62 (0.47)
*t* value	0.29	10.37***	0.62	0.56	−0.87	1.32

*SE*: standard error.

* *p* < .05; ***p < .001.

**Table 8.** One-sample *t*-test results for each condition and time window where there was a significant effect of condition.

	*df*	*t* value	*p* value
Ambiguous noun			
Adult			
Open	16	2.51	.02*
Secret	16	−1.87	.08
Adolescent			
Open	34	1.71	.10
Secret	34	1.41	.17
Transitive verb			
Open	51	2.73	.009**
Secret	51	−1.21	.23
Disambiguating noun			
Open	51	6.86	<.001***
Secret	51	−8.62	<.001***

*df*: degree of freedom.

* p < .05; **p < .01; ***p < .001.

Analyses during the ambiguous noun (e.g., “car”) showed no significant effect of Age group (all *p* > .22), and a nonsignificant trend for Context (*p* = .052). Crucially, however, there was a significant interaction between Context and Age Group 1 (adult vs. both adolescent groups) in this time window. Follow-up analyses tested the effect of context in each age group and revealed significantly different biases in Open and Secret contexts in the adult group (Est. = 0.92, *SE* = 0.31, *t* = 2.97, *p* = .005), reflecting a significant bias to fixate the open referent within an open context, and a nonsignificant trend to fixate the secret referent within a secret context (see Table 8). In contrast, the adolescents (young and old combined) showed no difference between the two context conditions (Est. = 0.07, *SE* = 0.25, *t* = 0.28, *p* = .778) and no significant bias to either referent during secret or open trials (Table 8). The Context × Age Group 2 (younger vs. older adolescent groups), interaction was not significant in this time window, showing that visual biases were equivalent between younger and older adolescents.

Analyses in the post-ambiguous noun (“and he”) and adverb (“deliberately”) time windows showed no significant effects or interactions. However, by the transitive verb (“chose”), analyses showed a significant effect of context, but no effects of Age group or any significant interactions. The post hoc *t* tests indicate that here, all participants showed a significant bias to the open referent within an open context but did not show a preference between open and secret referents in the secret context (see Table 8). During the disambiguating noun time window (“pink/green car”), there was a significant effect of context and no other effects or significant interactions (all *p*s > .24). Post hoc *t* tests revealed that all participants showed significant visual biases to the appropriate referent (i.e., the open referent during the open context and the secret referent during the secret context).

To summarise, the eye-movement data suggest that adults successfully used contextual information about the character’s higher order desires to distinguish open and secret actions as soon as they heard the ambiguous noun (e.g., “car”). They correctly anticipated reference to the open referent in the open condition (i.e., the pink car) but crucially, they also correctly anticipated reference to the secret reference in the secret condition (i.e., the green car). This suggests that adults not only showed a sensitivity to the different contexts but used that information about the character’s intentions to make predictions. The weaker effect found in this adult group during the secret trials is consistent with results from Ferguson & Breheny (2012). However, neither adolescent group seemed to show sensitivity to the context until much later, during the transitive verb (“choose”) which precedes the disambiguating words, suggesting that they do not make inferences about the perspective of the characters early on, like adults do.

#### Individual differences results

Results for one-way ANOVAs of all individual measures are shown in Table 9. The three age groups did not differ in either the simple or complex IC measures (all *p*s > .21). However, the Digit Span task revealed a significant difference in WM between age groups, with Bonferroni-corrected post hoc comparisons revealing that younger adolescents performed worse than older adolescents (*p* = .05) and adults (*p* = .003), but that older adolescents and adults did not differ (*p* = .89).

**Table 9.** Means and standard deviations (*SD*s) of all executive function measure scores and their respective one-way ANOVA results.

	Mean (*SD*)			ANOVA	
	Adult	Older adolescent	Younger adolescent	*df*	*F*
Inhibitory control					
Simple Go NoGo (Acc)	−0.05 (0.72)	−0.08 (0.14)	−0.11 (0.13)	2, 49	0.95
Complex Go NoGo (Acc)	−0.21 (0.16)	−0.24 (0.15)	−0.32 (0.22)	2, 49	1.59
Simple Go (RT)	387 (19)	383 (47)	403 (56)	2, 49	1.01
Complex Go (RT)	429 (50)	425 (53)	453 (72)	2, 49	1.14
Working memory					
Digit Span	8.00 (2.26)	7.28 (1.96)	5.59 (1.80)	2, 49	6.40**

ANOVA: analysis of variance; *df*: degree of freedom; Acc: accuracy; RT: response time.

** *p* < .01.

**Figure 6.**
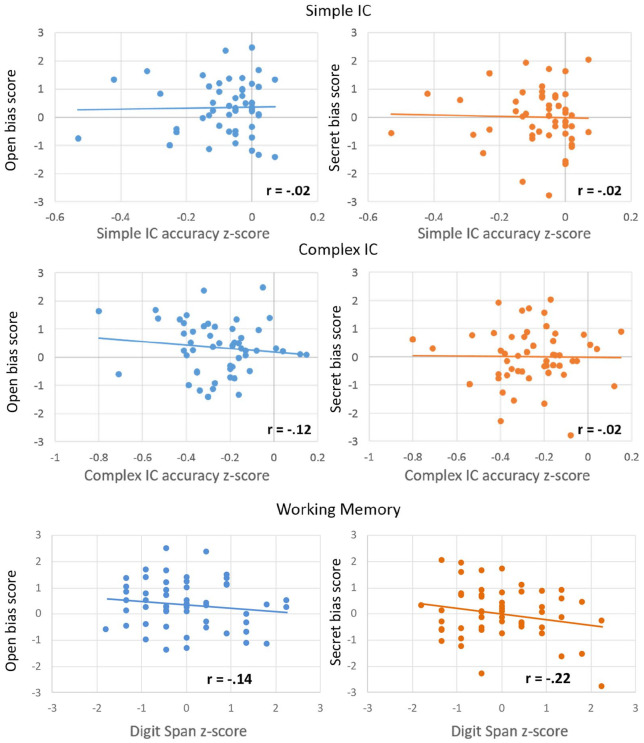
Correlations between participants’ anticipatory bias score (*y*-axis) and scores from each individual measure (*x*-axis), separately for the open (blue) and secret (orange) condition.

To further examine whether individual differences in IC or WM had an effect on anticipatory looks to the correct target, and whether this differs depending on whether the character intended to be open or secretive about their personal preference, Pearson’s correlations were performed separately for each condition between the average bias score during the ambiguous noun and participants’ computed *z*-scores for IC (simple and complex accuracy) and WM. Correlations were conducted for the ambiguous noun region (Figure 6) because this is where the first evidence of context effects emerged in this study and Ferguson and Breheny (2011), and age-group differences were found here. In addition, we conducted correlations for the entire anticipatory window (from the ambiguous word until onset of disambiguating word). As in Experiment 1, we did not run correlations using the median Go response times because responses on Go trials do not require IC. The significance level for each test was corrected for the six correlations using Bonferroni correction (*p*_crit_ = .0083). Results showed that there were no significant correlations for any of the individual measures and the bias score in either condition in the ambiguous noun region (all *p*s > .11) or the entire anticipatory region (all *p*s *>* .37).

### Discussion

Results from Experiment 2 showed that adults anticipated the appropriate target long before disambiguating information was presented (i.e., in the ambiguous “car” region) in both the *open condition*, when there was no conflict between the character’s basic preferences and high-order desires, as well as the *secret condition*, when there was a conflict between the character’s basic preference and high-order intentions. This pattern is in line with Ferguson and Breheny’s (2011) findings. Crucially, younger and older adolescents showed a delay in anticipating the target in both conditions, as distinct gaze biases for open and secret conditions only emerged in the region immediately preceding the disambiguating information (i.e., during “chose”).

Additional analyses of individual difference characteristics showed that although the younger adolescents had poorer verbal WM than the older groups, there was no age-related differences between older adolescents’ and adults’ IC and verbal WM performance. Thus, older adolescents were delayed relative to adults at anticipating the character’s actions despite comparable IC and verbal WM. None of the individual measures correlated with anticipation, reflected in eye-gaze data.

## General discussion

In this article, we have presented two visual-world studies involving adolescent and adult groups. The studies were designed to measure incremental formation of participants’ expectations about a character’s actions, given background information about their beliefs and desires. The studies differed in terms of the social complexity of the scenarios, reflected in the order of belief-desire reasoning required. In our first experiment, the character acts on a simple first-order belief about an object’s location, which could be true or false. In our second experiment, the character is portrayed as having a first-order preference and a second-order preference about what other people believe about that preference (i.e., a third-order mental state representation). Conditions differ in terms of whether the character prefers people to know about the first-order preference, or not. In both experiments, we successfully replicated previous findings with adult groups (Ferguson, Apperly, et al., 2015; Ferguson & Breheny, 2011). In both cases, adults were found to show sensitivity to a character’s mental states when anticipating their actions, in the earliest time region (i.e., they distinguished expectations for the character’s behaviour based on TB and FB in Experiment 1, and based on first-order desires and an intention to deceive in Experiment 2).

Previous visual-world research has examined incremental anticipation of others’ mental states in older adolescents versus adults using a version of the director task, the triples task. This task is well known to be challenging even for adults, whereas other versions of the director task are less so. Specifically, the triples task compounds the “pull of reality” demands imposed by other versions of the director task, with an additional demand of ignoring attraction to the distractor object due to “bottom up” linguistic processes (Heller et al., 2016). Previous comparisons have shown that adolescents, including older adolescents, are less likely to take the perspective of the director into account than adults. Differences in outcomes between older children and adolescents, and adults on the triples director task appears to be partly accounted by differences in EF abilities, which are required to a larger degree in the triples director task (Symeonidou et al., 2016). A change-of-location FB task, like less demanding pairs versions of the director task, requires only that participants ignore the “pull of reality” and hence poses lesser EF demands. Our aim with our first experiment was to determine whether, given the lower demands of a change-of-location FB task, but comparable first-order mental state inferences, adolescents would show anticipation in the same timecourse as adults. Experiment 1 showed that, in fact, adolescents’ gaze discriminated between TB and FB conditions more robustly than adults in early time regions (i.e., the pre-object region).

Our assumptions about the stimuli in Experiment 2’s secrets task was that EF demands are relatively low, in spite of the social complexity of the scenarios being described (Ferguson & Breheny, 2011). The results of the second experiment support this assumption. Here, we found no correlation between either measure of IC or verbal WM and anticipation. In particular, there was an absence of correlation even in the “secret” condition, where the character’s first-order and higher order preferences were in conflict. This absence is consistent with widespread thinking about previous ToM research, which sees EF demands stemming from factors like the “pull of reality” (Birch & Bloom, 2007; Ferguson & Breheny, 2012), response demands (Setoh et al., 2016; Wang et al., 2016), or interference from bottom-up linguistic processes (Hanna et al., 2003; Heller et al., 2016). Although these factors have been argued to affect outcomes in FB and director tasks, none are present in the secrets task.

Participants in Experiment 2 were presented with a character who has a preference that is potentially less than socially acceptable. This character is either untroubled about other people knowing about that preference or does not want other people to know. The visual-world paradigm used in this study is a useful way to measure the timecourse in which participants can integrate the incoming linguistic stimulus with background contextual knowledge to generate expectations about upcoming discourse. Replicating previous results, we found that our adult group showed clear anticipation of the likely course of events at the earliest possible point in the linguistic input. The adolescents also showed some anticipation of the character’s response before disambiguation. These results therefore provide evidence for an ability to make mental state inferences based on complex desires and intentions in late childhood and adolescence.

However, neither adolescent group displayed anticipation until much later in the target sentence. We can conclude from these results that the younger age groups are delayed in predicting outcomes, compared with adults. Based on a comparison between the older adolescent group and adults, we cannot directly attribute these differences to differences in EF abilities. Although we took into account differences in WM and IC, adolescent and adult groups may differ in other ways that may have affected their performance on the task, which is a limitation of this research. For example, performance of adolescents and adults on reasoning tasks can be differently affected by the presence of an experimenter (Wolf et al., 2015), and language development continues throughout childhood, adolescence, and adulthood (Nippold, 2006). Moreover, these more socially complex scenarios may lead to more uncertainty for adolescents, in terms of what they expect the character to do. One possibility is that adolescents and adults differ in the organisation and availability of task relevant information. For example, adults have richer networks of associations, and clearer biases, given social contextual cues. Another possibility is that adolescents generate less well-defined outcomes for scenarios than adults, due to differences in weightings of prior probabilities (buying a pink car vs. green), and uncertainty about background mental states, given observed behaviour.^5^ Viewing the results in these terms, future research could examine to what extent adolescents are able to appreciate that others’ behaviours often result from complex, conflicting motivation. The development of this ability to deal with conflicting desires and mental states may parallel the prolonged development of the understanding and experience of mixed emotions (e.g., Burkitt et al., 2019; Larsen et al., 2007).

This finding of a developmental difference in mental states inference between adolescents and adults is in line with neuroimaging studies showing continued development of the “social brain” during adolescence (Blakemore, 2008). Our results indeed reveal that ToM has not reached full maturity by adolescence; younger and older adolescents were delayed relative to adults in anticipating the target in scenarios that required complex mental state reasoning (Experiment 2). Importantly, although previous research on adolescent social development has found that EF abilities can be a significant factor in accounting for differences between children, adolescent, and adult groups on tasks that call on ToM (Mills et al., 2015; Symeonidou et al., 2016), the current age groups differences were not accounted for by difference in simple or complex IC, or verbal WM. Our findings therefore are strong evidence of prolonged maturation of social reasoning about complex mental states.

Nevertheless, we acknowledge the potential limitation of sample size; we simply may not have had sufficient power to accurately detect the age group × belief/context interaction effects in our experiments. In this study, detecting a significant interaction with the significance level of α = .05 on 80% of occasions (as suggested by J. Cohen, 1988) would have needed a minimum of 90 participants for Experiment 1, and 100 for Experiment 2 (calculated using the *simr* package in R; Green & MacLeod, 2016). It would have been very challenging to recruit and test these high numbers of participants, using the complex eye-tracking methods we used. The current sample sizes yield an estimated power of 41% for Experiment 1 and 51% for Experiment 2. Although the lower sample sizes are somewhat mitigated by the use of linear mixed models where analyses control for both by-participant and by-item variation rather than aggregated across participants (thus improving power; Baayen, Davidson, & Bates, 2008), the power calculations noted above are based on this analysis approach and so the study is underpowered. Nevertheless, the results in the adult group broadly replicated the patterns seen in the experiments that we sought to replicate (Ferguson & Breheny, 2011; Ferguson, Apperly, et al., 2015), meaning that we can feel relatively confident that the reported findings are reliable. As a field, research on adolescent cognitive development should continue to aim for larger sample sizes.

Regarding the EF demands of the tasks reported in this article, when correcting for multiple comparisons, we found no significant correlation overall between performance on the complex-IC task and the FB trials in Experiment 1. We note, however, that previous research finds that change-of-location FB tasks pose a “pull of reality” challenge due to the location of the mentioned object being different to the target location (e.g., Apperly et al., 2008; German & Hehman, 2006). It is interesting that none of the EF demands in this FB task resulted in a change of outcome, in terms of the timecourse of anticipation, between adolescents and adults. We can attribute this to the fact that demands of FB tasks are not that great, compared perhaps with the more demanding triples versions of the director task. Whether a no-difference outcome would emerge between groups whose EF scores actually differ is a question we leave to another time.

Moreover, results of Experiment 2 provide very clear evidence that factors outside of EF differences can lead to differences in outcomes between groups of adolescents and adults. As mentioned, we found no relation between anticipation and any EF measure. The older adolescent group in this experiment did not differ to adults on these measures, and yet outcomes were markedly different. These results are in line with views emerging in the literature on older children and adolescents that there is more to social cognitive development than simply differences in EF (Bradford et al., 2020; Brunsdon et al., 2019; Dumontheil et al., 2010; Zhao et al., 2018). Our results indicate ways forward for adolescent social-cognitive research which are sensitive to EF abilities but which probe responses to more complex events, and the biases and expectations that may underpin those responses.

## Notes

1. In both the pairs and triples versions of the director task, participants hear a referring expression with a modifier (e.g., “the small candle”). In the triples version (e.g., Keysar et al., 2000), the target object is the smaller of two candles in common view but it is not the smallest candle visible to the participant, as there is an even smaller candle which the participant but not the director can see. Several replications of this study show that even adults find it difficult to ignore the best-fit competitor and even reach for that object (e.g., Dumontheil et al., 2010; Heller et al., 2016; Wu & Keysar, 2007). In the pairs version (e.g., Heller et al., 2008; Nadig & Sedivy, 2002), the participant sees a small (target) candle and a larger (target contrast object) candle, as well as a small cup (competitor) and either a larger cup (competitor contrast object) or irrelevant distractor. When all the participant’s information is in common view, it is shown that participants anticipate the target while hearing the modifier (“small”) when the competitor contrast object is absent (Sedivy et al., 1999). When the competitor contrast object (large cup) is only visible to the participant, the prediction is that the same advantage in anticipating the target would occur if the participant can accommodate the director’s ignorance of this object. This is what is found (Heller et al., 2008, 2016; Nadig & Sedivy, 2002).

2. Note that a post hoc power calculation showed that a minimum of 90 participants (calculated using the simr package in R; Green & MacLeod, 2016) would have been needed to detect a significant two-way interaction with the significance level of α = .05 on 80% of occasions (as suggested by J. Cohen, 1988). The current sample sizes yield an estimated power of 41% for Experiment 1. We discuss this limitation further in the “General discussion” section.

3. Note that no visuospatial working memory (WM) measure was collected in this study. Visuospatial WM continues to develop during adolescence (e.g., Conklin et al., 2007; Dumontheil et al., 2011) and may help participants track the movement of the characters and objects in the false-belief task used in this study. So any age group differences may be driven by possible differences between the groups in visuospatial WM capacity.

4. Note that a post hoc power calculation showed that a minimum of 100 participants (calculated using the simr package in R; Green & MacLeod, 2016) would have been needed to detect a significant two-way interaction with the significance level of α = .05 on 80% of occasions (as suggested by J. Cohen, 1988). The current sample sizes yield an estimated power of 51% for Experiment 2. We discuss this limitation further in the “General discussion” section.

5. For example, a Bayesian model of a participant’s expectation for an outcome where the agent buys the green car (*o*_g_) or a pink car (*o*_p_), given either “out and proud” or “secretive” context, *C*_o_ or *C*_s_, might see the distribution as a function of the likelihood of *C*, given observed behaviour and the prior probability as follows:

P(o\|C)μP(C|o).P(o).

Successful anticipation of the more socially acceptable green-car choice, given a “secretive” context, would stem from an awareness both of a greater prior probability of socially acceptable behaviour, *P*(*o*_g_) > *P*(*o*_p_), and of a sensitivity to the fact that observed behaviour often results as a compromise between complex, conflicting desires, *P*(*C*_s_|*o*_g_) > *P*(*C*_s_|*o*_p_). This discussion assumes a crudely simple analysis of a more complex state of affairs. See Goodman et al. (2006) and Baker et al. (2009) for discussion.
